# What Is Physical Activity? A Holistic Definition for Teachers, Researchers and Policy Makers

**DOI:** 10.3389/fspor.2020.00072

**Published:** 2020-06-18

**Authors:** Joe Piggin

**Affiliations:** School of Sport, Exercise and Health Sciences, Loughborough University, Loughborough, United Kingdom

**Keywords:** physical activity, health, definition, interdisciplinary, sport, exercise, fitness

## Abstract

This conceptual analysis presents an argument that a new and broader definition of physical activity is needed for educators, researchers, and policy makers. To build a case for change, this paper has four parts. First, it outlines why definitions are important. Second, the current dominant definition of physical activity is examined and critiqued. Third, the case for change to the dominant definition is made. Fourth, a new, broader definition for physical activity is offered and justified. The new, broader definition of physical activity is proposed as involving “people moving, acting and performing within culturally specific spaces and contexts, and influenced by a unique array of interests, emotions, ideas, instructions and relationships.”

## Introduction

Definitions in educational settings, research, and policy are important for various reasons. Definitions set boundaries on phenomena and processes. Definitions also inform policy. Choices about whether to intervene in a health or social problem depend on how the problem is framed and what measures are used to understand the problem. However, definitions can be contentious and confusing. In a review of literature, Frérot et al. (2018) found 102 (English) definitions of “epidemiology” and highlighted the evolving nature of the definition over time. Also recently, a Sedentary Behavior Research Network conducted a literature review to gather “any evidence of inconsistencies, differences, conflicts, or concerns over variations in definitions” of sedentary behavior and related terms (Tremblay et al., 2017). In aiming to produce a consensus definition of sedentary behavior, the research group found at least 12 definitions of “sedentary behavior” being used in the academic literature.

Rorty (1999) notes that we define the way we do “because of our needs and interests” (p. xxvi). In this vein, as a researcher involved in physical activity, sport studies, and health promotion, I have witnessed over the last decade increasing attention and interdisciplinarity in the area of “physical activity”. As such, it seems an opportune moment to critique some of the taken-for-granted ideas which inform and guide educational settings, research, and policy about physical activity. Schiappa (2003) argues that all definitions are linguistic propositions and as such are historically situated, “and the beliefs that inform definitions are human beliefs that are always subject to revision…” (p. 9). There is an apparent inadequacy in the existing dominant definitions of physical activity to account for its complexity. Therefore, this conceptual analysis presents a new, broader definition which might provide opportunities for physical activity to be understood in a more ethical and holistic manner. On this point, Schiappa (2003) notes there is space to consider the ethical and normative ramifications of the act of defining, and that problems faced by citizens might be better addressed with the acknowledgment that definitions are rhetorically induced social knowledge. And so what follows here is not an argument for the exclusion of traditional definitions of physical activity. However, by offering a change to the orthodox definition, teachers, students, researchers, and policy makers can reflect on the strengths and limitations of various definitions. Further, we might more appropriately connect the language we use with the physical activity we are concerned with.

## Physical Activity—the Predominant Definition

To contextualize the status quo regarding definitions of physical activity, I trace the origin and growth of the most widely accepted definition, published by Caspersen et al. (1985). They define physical activity as “any bodily movement produced by skeletal muscles that results in energy expenditure” (p. 126). This definition produces a very specific way of understanding physical activity. The focus on “skeletal muscles” and “energy expenditure” frames physical activity as a specific mechanistic act. This is illustrated by the emphasis immediately following the definition where the authors focus on how energy is measured:

The amount of energy required to accomplish an activity can be measured in kilojoules (kJ) or kilocalories (kcal); 4.184 kJ is essentially equivalent to 1 kcal (1). Technically, the kJ is preferred because it is a measure of energy expenditure; however, historically the kcal, a measure of heat, has been employed more often (pp. 126-127).

This definition is widely used and accepted within the research community. The article by Caspersen et al. (1985) has been cited 9490 times in Google Scholar (at the time of writing), an indication of its popularity. This definition informs many health policies around the world (Australian Government Department of Health, 2011; World Health Organisation, 2018; UK Chief Medical Officers, 2019), as well as academic textbooks (Biddle and Mutrie, 2001; Hardman and Stensel, 2003), and journals (Howley, 2001; Haseler et al., 2019). There does not appear to have been published analysis or critique of this dominant definition of physical activity, except for some small variations of the definition, explained below.

## Small Variations of the Definition

There are small variations on this definition. In 2018, the World Health Organisation's (WHO) Global Strategy on Physical Activity deployed a slight variation of Caspersen's definition. Instead of activity *resulting* in energy expenditure, the WHO referred to bodily movement that “*requires* energy expenditure” (2018, p. 14).

Variations can also occur by the same author. The US Surgeon General's report (U. S. Department of Health Human Services, 1996) defined physical activity as

bodily movement produced by the contraction of skeletal muscle that *increases energy expenditure above the basal level* (p. 20, italics added).

However, on the next page of their report in a glossary, physical activity was defined as:

bodily movement that is produced by the contraction of skeletal muscle and that *substantially increases energy expenditure* (p. 21, italics added).

Sometimes slight additions are present. In 1995 the US National Institutes of Health (NIH) Consensus Statement inserted “health benefits” into the definition of physical activity:

“bodily movement produced by skeletal muscles that requires energy expenditure' *and produces healthy benefits*” (National Institutes of Health, 1995, p. 3, italics added).

The idea that all physical activity produces healthy benefits is open to critique, since activities such as overtraining, repetitive strain, and physical combat might all count as physical activity but do not necessarily produce health benefits for all. Any definition should therefore avoid absolute claims about the benefits of physical activity on health promotion.

I argue here that these small variations to the 1985 definition all focus on bodily movement, skeletal muscles and energy expenditure. Among them all, the sentiment remains the same. The 1985 definition, and the small variations of it are confined to, and thereby constrained by, epidemiology discourse. Indeed, the introductory sentence by Caspersen is “The epidemiological study of any concept or event requires that the item under investigation be defined and measured” (p. 126). By describing the “elements” of physical activity, the focus is on “bodily movement, skeletal muscles, energy expenditure, kilo-calories” and a positive correlation with “physical fitness” (p. 127). Consequently, the definition proposed by Caspersen is heavily laden with biomedical values, to the exclusion of much else. While Caspersen's definition of physical activity may be appropriate for certain epidemiological studies, it does not do justice to physical activity outside of that specific domain.

## The Case for Change

What follows is an attempt to promote definitional “rupture” (Schiappa, 2003). The hope is to move the definition of physical activity from its entrenchment in epidemiological and biomedical discourse and toward a more inclusive, holistic usage which accounts for the complex nature of physical activity. However, any new definition should also be accessible and useful to those involved in epidemiology. There is an opportunity to open up the definition of physical activity to be more inclusive for many groups, including the academic disciplines that study it, the governmental departments that write policy on it, and the range and depth of human experiences which both produce and are produced by it.

### The Narrowness of the Popular Definition

What follows is a critique of the Caspersen et al. (1985) definition: physical activity as “any bodily movement produced by skeletal muscles that results in energy expenditure.”

The definition must first be situated in the specific scientific context and rationale for its creation. The title of Caspersen's article is “Physical Activity, Exercise, and Physical Fitness: Definitions and Distinctions for Health-Related Research”. As such, the terms are situated in a specific domain (health-related research), and the article aims to “distinguish” between the terms because “they are often confused with one another, and the terms are sometimes used interchangeably” (p. 126). The authors' rationale may have been thoroughly reasonable, though it is important to recognize the discursive boundaries and disciplinary limitations that might be imposed with such aims. By focusing on “health-related research and epidemiology,” other inherent aspects of activity such as cognition, physical literacy, social cohesion, and education, are not accounted for. These aspects will be discussed in more detail later.

Caspersen et al. suggested that distinguishing between physical activity, exercise and fitness would assist “as an interpretational framework for comparing studies” (p. 126). While this may have been a worthwhile endeavor, the definition has been deployed beyond the realm of study comparison and has become established as the most popular definition of physical activity. It is possible of course, but unlikely, that the authors intended the definition for either policy statements, or as a broad, inclusive definition for physical activity. However, the dominant use of the narrow definition means that there is no space to account for the complex, holistic elements of physical activity.

Indeed, Caspersen et al. acknowledge the disciplinary boundaries within the article by stating that “The epidemiological study of any concept or event requires that the item under investigation be defined and measured” (p. 126). The concern of epidemiologists to prevent disease and the methodological orthodoxy governing the types of knowledge which epidemiology has produced is worth considering. The British Medical Journal supports a definition of epidemiology as “the study of how often diseases occur in different groups of people and why” (Coggon et al., 2003). By framing physical activity in relation to disease-potential and disease management, much is marginalized and ignored. To illustrate this point, I offer how Pronger (2002) was troubled by the contrast between his experience of his active childhood and the “technological knowledge” within his university studies in physical education:

“I wrote about ‘the powerful source,' the wonder and infinity that I discovered in swimming. And I said that when I started to study physical education, that dimension was completely absent from everything we were taught. The technological education that I was receiving rendered the wonder second. And as I survey the array of scientific, government and commercial texts on physical fitness, I hear only silence in this regard. The technology of physical [fitness] seems deaf to this dimension of life. So the question of secondness here is: what kind of life is produced in such deafness? But another question also arises: what latent possibilities does that silence hold?” (p. 15)

The reason why the Caspersen et al. definition is still used as the dominant and widely accepted definition is due in part to its simplicity and clarity. Thus, there is an opportunity to examine what is silent in the dominant definition of physical activity. There is space to acknowledge health aspects of physical activity while also emphasizing its complex and multifaceted aspects in a new, broader definition.

The simplicity of the Caspersen definition also belies what is omitted. Each component of the definition will be critiqued for what it limits or omits. First, the phraseology of “any bodily movement” may be useful in a clinical setting, but it unwittingly depersonalizes activity. Second, the idea that movement is “produced by skeletal muscles” also limits the domain of investigation to distinctly narrow biomechanical characteristics, instead of being produced by an agentic, motivated human. Third, the argument that physical activity “results in energy expenditure” omits all else that can result from, be produced by, or created through physical activity. Therefore, Caspersen's et al. definition is dis-integrated and exclusionary because it emphasizes some elements—the anatomical and physiological, over others. And so with this context in mind, there is space to create a more expansive, inclusive, holistic definition of physical activity, which might inform not only its scientific study, but also contribute to policy statements, the framing of interventions in populations, and the teaching of the topic in physical activity, physical education and health educational settings.

## Inherent Aspects of Physical Activity

The following considerations are used to argue what physical activity inherently involves and which aspects should be emphasized or included in a definition. While I accept the discussion by Caspersen regarding what occurs at the “physiological level” during physical activity, there are numerous inherent qualities of physical activity that need to be acknowledged to more fully express what physical activity is.

### Physical Activity Is Inherently Cerebral

Discussions of the mind use different terms—cerebral/cognitive/psychological/emotional/affect, and so on. In any case, physical activity is so innately intertwined with the human mind as an antecedent (or motivator) of activity, as the central processor of the experience, and as being responsible for remembering and reflecting on the experience, that to exclude it from a definition renders it incomplete. Biddle and Mutrie (2001) note that:

“a great deal of physical activity for health must be freely chosen in leisure time or consciously integrated into one's normal daily routine. This, in itself, justifies the increasing importance of studying psychological processes, such as motivation and decision making, in physical activity” (p. 7).

Psychological theories to explain physical activity behavior abound. Furthermore, from the COM-B theory, to the “behavior change wheel,” to nudging theory (Forberger et al., 2019), psychological theories of motivation have been used to promote physical activity through interventions at a population level (Brand and Cheval, 2019). Psychology therefore is the intervention point to inspire or produce physical activity. Inherent psychological components deserve recognition as part of physical activity as much as (if not more than) the spending of energy. Policy texts do increasingly mention ideas about mental well-being, but they tend not to stray into aspects of “wonder,” as discussed by Pronger (2002). A holistic definition will move beyond “bodily movement” to incorporate, appreciate, and celebrate the lived experiences that produce physical activity.

Physical activity is also a deeply affective, emotional activity. The spectrum of emotions in physical activity range from joy and feelings of empowerment that can come from active games (Light, 2003), to the potential for humiliation and anguish for participants in physical education (Sykes and McPhail, 2008). The 1985 definition never mentions cognition or emotion. Appreciating the full range and significance of the emotional aspects of physical activity is therefore imperative to understand physical activity in a rounded fashion.

### Physical Activity Is Inherently Social

Whether it is Oxford and Cambridge oarsmen (Hartley and Llewellyn, 1939), Cambridge sportmen (Rook, 1954), or London busmen (Heady et al., 1961), physical activity is an inherently social (and clearly gendered) activity. As social beings, humans move through space in communion with others (such as in protest marches), in competition with others (in sport), out of necessity (for food gathering or employment) or for pleasure (sexual, cathartic or otherwise). These endeavors result in an array of productive, creative outputs, which should not be underestimated in comparison with the health benefits that tend to dominate academic discourse on physical activity. For example, Bairner (2012) noted that the health gains of walking “may well be of secondary importance to the lessons that can be learned from the pedagogies of the street” (p. 373).

### Physical Activity Is Inherently Situated

It is well-established that physical and cultural spaces shape experiences (Phoenix and Bell, 2019). The ways in which these settings can be described are numerous. Urban-rural, natural-cultural, wild-managed, poor-wealthy, and numerous other varieties of spaces and contexts produce both opportunities and barriers to the types of physical activities that are possible (Collins and Kay, 2014). In turn physical activity shapes spaces. There is a symbiotic relationship between people, activities and spaces (Cherrington and Black, 2020).

In the article by Caspersen et al. (1985), the authors do mention that while “the simplest categorization identifies the physical activity that occurs while sleeping, at work, and at leisure,” it is also a “complex behavior … and may be meaningfully partitioned into other categories mutually exclusive of each other” (p. 127). This may be appropriate for measuring energy expenditure (as that was the emphasis of the article) but there is a disjuncture between the idea of mutual exclusivity of categories and the now apparent messy interplay between all manner of pressures and influences on physical activity. This can be seen in the growing popularity of systems thinking and ecological approaches to understand physical activity. These approaches situate physical activity as taking place in, and affected by, a wide variety of cultural values, economic conditions and physical settings. Rutter et al. (2019) offer a preliminary analysis of the “drivers of physical activity,” which might be either synergistic or antagonistic in the production of physical activity. The growing range of systems theories shed light on the complex issues which shape, and in turn are shaped by, physical activity.

### Physical Activity Is Inherently Political

Politics shape the provision and structure of physical activity. This occurs at many levels, from state resources for public spaces, to traditional ways that physical activity is provided or promoted. The “political” can also include efforts involved in controlling and judging the activities that people partake in. Therefore, more depth, richness and inclusivity might come from redefining physical activity to account for its complexities, nuances, and politics. Writers in physical cultural studies argue that human movement can and should be considered from a variety of levels, including “the socio-cultural, discursive, processual, institutional, collective, communal, corporeal, affective, and subjective” (Silk et al., 2017, p. 1). In parallel to academic discussions, it is apparent that numerous ideas inform state physical activity promotion including and aside from public health, such as environmental sustainability and education (see UK Government, 2019). Making claims about the importance of some reasons over others is an inherently political act, which requires value judgements about the legitimacy and relative importance of desired benefits.

By accounting for such depth in a new, broader definition, we can expand both the thinking about physical activity and policies which are written for it. By changing the words used to construct a definition (to include emphasis on the social, psychological and political), we can remove the narrow confines of epidemiological discourse. Further, by examining the reasons for promoting various ideas, we can critically reflect on motivations that may or may not be in the interests of those targeted by policy interventions (see Piggin, 2015). Examining the politics of physical activity involves asking various questions. Which ideas gain prominence and are emphasized in eventual decisions? Whose ideas are marginalized and omitted from policy discussions? (Piggin, 2019). Policy decisions (and non-decisions) about physical activity contribute to the dignity, values and life chances of individuals and communities. The rules and values which allow and influence activity should be critically appraised, especially since all people are subjected to evaluation and judgements of what are “culturally appropriate” activities (see World Health Organisation, 2018). Physical activity involves an interplay between external factors and internal perspectives, sensibilities, and motivations. This interplay should be acknowledged in a holistic definition.

## What is Physical Activity? a new, Broader Definition

Proposing a new, broader definition of physical activity disrupts the current reductionist (simplistic) explanation of physical activity in favor of emphasizing the holistic (complex) nature of it. Given the orthodoxy that the Caspersen et al. definition has established, such a disruption might also be very useful. Expanding the definition may illuminate new ways of thinking about physical activity and open up diverse ways of teaching, researching and making policy for physical activity. Academically, it emphasizes interdisciplinarity and inclusion, and provides opportunity to question, critique, celebrate, and create new ways of talking about and thinking about physical activity. Related to this, questioning the orthodoxy may also be met with significant resistance. Many users of the Caspersen definition may ignore challenges or indeed defend the status quo.

Understanding more about one's own activity might also be a benefit of a broader definition. One justification for this is to allow the *lived experiences of people* to be recognized. The aim is to move beyond the boundaries of epidemiological discourse or disease prevention and toward an acknowledgment of the dynamic, complex, and evolving array of reasons and emotions involved in physical activity. The new and broader definition is provided below:

***Physical activity involves people moving, acting and performing within culturally specific spaces and contexts, and influenced by a unique array of interests, emotions, ideas, instructions and relationships*.**

This definition was first introduced in the book The Politics of Physical Activity (Piggin, 2019), though this is the first time that the various justifications are explained in detail. There are numerous benefits of an expanded definition.

First, it prioritizes *people moving* over muscles moving. Of course, this change does still accommodate kinesiologists. Focusing on people moving does also include biomechanical and physiological aspects of activity. However, for the aim of inclusivity, it redirects attention to the *person* rather than *skeletal muscles* or *energy as kilojoules* in the first instance. An expanded definition *emphasizes complexity, the environment and the human experience*. Accommodating the cognitive, affective and situated aspects of physical activity will allow users and teachers of the definition to account for the complexity of physical activity (see Pronger, 2002). The inclusion of social and cultural contexts and the array of influences allows for the consideration of opportunities and constraints to physical activity.

Second, by discussing *acting and performing* as well as moving, the definition appreciates the productive and creative potential that comes from physical activity. Distinct and in contrast with the original definition's emphasis on energy *expenditure*, the new definition imagines that much more is *created* through physical activity (such as the outcomes of labor, artistic performances and emotional, memorable experiences) than spent. By shifting away from a focus on exertion (measured by technical apparatuses) we can more appropriately acknowledge and appreciate the range of other reasons for people being active.

Third, by emphasizing *inclusivity, complexity*, and *the holistic*, we can problematize the dualism (separation of the mind from the body) which emanates from Caspersen's original definition. Questioning dualism allows the reader to move away from a discourse of the “body as machine” and incorporate ideas about the “body as self” (see Whitehead, 2001). As such, a new, broader definition may be particularly useful for introductory university classes on physical activity, across a variety of disciplines.

Fourth, a new, broader definition might be useful in reframing policy interventions, beyond disease risk as a justification. This is not intended to marginalize the medical aspects of physical activity, though it is intended to resist against *over-medicalization*. This definition might open new ways of talking about activity, particularly within a policy sphere. For policy makers, it might elevate rights and values associated with physical activity to a higher priority, rather than the health benefits of physical activity remaining as the dominant justification for physical activity interventions. That is, there is more to health than physical activity, and there is more to physical activity than health. It might also stimulate novel ways of thinking about the place and meanings of physical activity for different people and different sectors of society. As the starting point for research studies it might provide impetus toward more inclusive questions and settings for research to take place.

Fifth, when people move they are influenced by a unique array of *interests, emotions, ideas, instructions, and relationships*. By acknowledging and prioritizing this, users of the definition can consider the wide range of intrinsic and extrinsic factors that are “unique” to each person's experience of physical activity. These interests, emotions, ideas, instructions, and relationships (which I argue are omnipresent before, during and after physical activity) might well be marginalized when there is a focus on “energy expenditure.”

There are parallels between the definition presented here and other disciplines. For writers on physical literacy (who themselves define physical literacy in various ways), the concern is often around “the motivation, confidence, physical competence, knowledge, and understanding to maintain physical activity throughout the life course” (Whitehead, 2013). Interestingly, one systematic review of physical literacy deferred to the WHO definition of physical activity and suggested a relation between the two concepts (Edwards et al., 2017).

## Discussion

A proposal for a new, broader definition of physical activity might be discomforting for some users of the Caspersen et al. (1985) definition. However, the variety of benefits from an expanded definition, coupled with the increasing interdisciplinarity of physical activity in the academic and policy spheres, indicates a more inclusive definition is important (see Table 1). Physical education, physical literacy, and physical cultural studies are all disciplines which have attempted to confront the complexities inherent within them to forge newer, more useful definitions. There is no apparent reason why the domain of physical activity has not seen a flourishing variety of definitions. The Caspersen et al. definition does not seem to have been subject to critical scrutiny in the past. Possible reasons include an epidemiological community that is largely satisfied with the definition or a lack of need or desire to account for the holistic nature of physical activity.

**Table 1:** Elements of the Caspersen et al. (1985) definition, compared with the Piggin definition (2019).

**Caspersen et al. (1985)**	**Piggin (2019)**
Bodily movement	People moving Acting Performing
Skeletal muscles	Culturally specific Spaces Contexts
Results in energy expenditure (kilojoules)	Influenced by Interests Emotions Ideas Instructions Relationships
Positively corelated with physical fitness (note: this element did not appear in the original definition, though appeared in a separate table)	

While the definition presented here advances the conversation about what physical activity is, the author does not claim definitional certainty. Indeed, rather than advocating for immediate consensus around this definition, a plurality of definitions is welcomed and encouraged, particularly since by doing so, more critical conversations can be held about what to include and what to leave out. Therefore, rather than consensus, it is hoped that this definitional disruption will open space for discussion and celebration of what can count as physical activity, what contributes to it (beyond calorific energy) and what is created by it. While Edwards et al. (2017) and Tremblay et al. (2017) advocated establishing a “consensus” for their definitions of physical literacy and sedentary behavior, respectively, this article argues for the opposite. There is a specific need for a definitional rupture to reframe physical activity to include the variety of inherent aspects that have traditionally been subjugated in favor of dis-integrated anatomical and physiological aspects. Moving away from reductive simplicity and toward wondrous complexity will likely contribute to a deeper appreciation and more nuanced understanding of physical activity.

## Author Contributions

JP conceptualized and wrote the paper.

## Conflict of Interest

The author declares that the research was conducted in the absence of any commercial or financial relationships that could be construed as a potential conflict of interest.
